# The Ratio of Dietary Branched-Chain Amino Acids is Associated with a Lower Prevalence of Obesity in Young Northern Chinese Adults: An Internet-Based Cross-Sectional Study

**DOI:** 10.3390/nu7115486

**Published:** 2015-11-18

**Authors:** Yan-Chuan Li, Ying Li, Li-Yan Liu, Yang Chen, Tian-Qi Zi, Shan-Shan Du, Yong-Shuai Jiang, Ren-Nan Feng, Chang-Hao Sun

**Affiliations:** 1 Department of Nutrition and Food Hygiene, School of Public Health, Harbin Medical University, Harbin 150086, China; liyanchuan2013@foxmail.com (Y.-C.L.); liying_helen@163.com (Y.L.); yanziliu2100@163.com (L.-Y.L.); liushn0505@163.com (Y.C.); ztq0211@163.com (T.-Q.Z.); dushanshan1007@163.com (S.-S.D.); 2 College of Bioinformatics Science and Technology, Harbin Medical University, Harbin 150086, China; jiangyongshuai@126.com

**Keywords:** dietary BCAA, overweight/obesity, abdominal obesity, 2 h-PG, inflammation

## Abstract

This study aims to examine the association between the ratio of dietary branched chain amino acids (BCAA) and risk of obesity among young northern Chinese adults. A total of 948 randomly recruited participants were asked to finish our internet-based dietary questionnaire for the Chinese (IDQC). Associations between dietary BCAA ratio and prevalence of overweight/obesity and abdominal obesity were analyzed. Furthermore, 90 subjects were randomly selected to explore the possible mechanism. Dietary BCAA ratio in obese participants was significantly lower than non-obese participants. We found negative correlations between the ratio of dietary BCAA and body mass index (BMI) (*r* = −0.197, *p* < 0.001) or waist circumference (*r* = −0.187, *p* < 0.001). Compared with those in the first quartile, the multivariable-adjusted OR (95% CI) of the 3rd and 4th quartiles of dietary BCAA ratio for overweight/obesity were 0.508 (0.265–0.972) and 0.389 (0.193–0.783), respectively (all *p* < 0.05). After stratification by gender, the significance still existed in the 3rd and 4th quartile in males and the 4th quartile in females. For abdominal obesity, the multivariable-adjusted OR (95% CI) of the 3rd and 4th quartile of dietary BCAA ratio were 0.351 (0.145–0.845) and 0.376 (0.161–0.876), respectively (all *p* < 0.05). This significance was stronger in males. Further studies indicated that dietary BCAA ratio was inversely associated with 2-h postprandial glucose (2 h-PG) and status of inflammation. In conclusion, a higher ratio of dietary BCAA is inversely associated with prevalence of obesity, postprandial glucose and status of inflammation in young northern Chinese adults.

## 1. Introduction

Branched-chain amino acid (BCAA), including leucine (Leu), isoleucine (Ile) and valine (Val), are essential amino acids for human beings. Previous studies reported that BCAA plays an important role in the process of protein synthesis, especially during periods of energy restriction and after physical exercise [1,2]. One study of wrestlers found that BCAA supplementation with energy restriction was more effective in reducing body fat than energy restriction alone [3]. Other studies reported beneficial effects from leucine [4] or protein (rich of BCAA) [5] on reducing body weight and body fat. Moreover, BCAA intake was shown to improve insulin resistance in mice [6] and humans [7], and might play an active role in the process of anti-obesity. However, most previous studies were limited to obese individuals or combined with special interventions such as energy restriction [3,4,5,8,9]. To our knowledge, only one population study based on a 24-h recall dietary survey suggested a negative association between BCAA intake and obesity [10]. However, overweight and obesity status in that study were defined as body mass index (BMI) ≥ 25 and BMI ≥ 30 kg/m^2^, which is not consistent with Chinese BMI cut-off points (≥24 kg/m^2^ for overweight status and BMI ≥ 28 kg/m^2^ for obesity) [11]; higher cut-off points may lead to lower estimated prevalence of overweight/obesity in the Chinese population. Secondly, that study was based on a 24-h recall dietary survey, which cannot represent long-term habitual food intake. Moreover, data on obesity-related metabolic parameters and cytokines are lacking. To our knowledge, no study has analyzed this association in the general population using a food frequency questionnaire (FFQ). Whether long-term intake of dietary BCAA is associated with obesity is still unclear.

Overweight and obesity are major risk factors for chronic diseases, such as coronary heart disease [12], diabetes [13], hypertension [14], dyslipidemia [15] and certain cancers [16]. During the past few decades, the prevalence of overweight, general obesity and abdominal obesity has increased world-wide [17]. Nutrition is important for prevention of obesity and reduction of excess body weight. Diets with higher protein and lower carbohydrate content have been shown to promote greater loss of body fat and reduced loss of lean body mass [18]. Therefore, reasonable dietary intervention should be useful for the prevention of obesity.

To investigate the dietary habits of a large population in a convenient way, we have designed and developed an internet-based dietary questionnaire for the Chinese (IDQC), which contains reference images of different weights of each food to aid in estimation of food intake. Compared with traditional FFQs, the IDQC provides a convenient and accurate tool to verify the hypothesis that the ratio of dietary BCAA intake is inversely associated with overweight/obesity and abdominal obesity in young northern Chinese adults. In this study, we examined the associations between dietary BCAA intake and prevalence of overweight/obesity and abdominal obesity among young northern Chinese adults using the IDQC. Moreover, a subgroup study was performed to explore possible mechanisms of the anti-obesity effect of BCAA.

## 2. Methods

### 2.1. Development of the IDQC

We designed a simple and convenient tool named IDQC for dietary data collection at Harbin Medical University, which was described and validated previously [19]. Briefly, commonly eaten foods were divided into 16 categories. Each participant had to choose the frequency and amount of each subtype of food groups, including: grains; potatoes; beans and their products; vegetables; fungus; fruits; seeds and nuts; livestock; poultry; dairy; eggs; fish; snacks; sweet foods; condiments; and beverages. The questionnaire was converted to hypertext markup language (HTML) format with option buttons for each question and was placed on a secure web server at net.cn (www.yyjy365.org/diet). To aid in the estimation of food portions, clickable images were created for each food as a reference. After completing the questionnaire, participants could obtain feedback on their dietary intake evaluation.

### 2.2. Reliability and Validity of the IDQC

The reliability and validity of the IDQC has been validated previously [19]. Briefly, 84 randomly selected students of Harbin Medical University were asked to finish the IDQC twice with an interval of 4 months. Dietary nutrient intakes were calculated and compared to examine the reliability of the IDQC. Moreover, an actual dietary record was kept for 3 consecutive days after the first time of IDQC and the correlation of the average nutrient intake in dietary record and IDQC was compared to examine the content validity of the questionnaire. Although the average nutrient intakes calculated by the IDQC were higher than actual dietary intakes, the positive correlation proved that the IDQC was a credible tool to estimate nutrients intake. Data were provided in supplemental materials (Tables S1 and S2).

### 2.3. Participants and Exclusive Criteria

Participants aged 18–40 were randomly recruited at the Health Examination Center of the Second Affiliated Hospital of Harbin Medical University from 1 February to 15 May 2014, through the serial number of the hospital. All selected participants were invited to register an account at www.yyjy365.org/diet. In order to ensure no repeat registration, the phone number of each participant was used as the account. A total of 1400 were invited, and 1177 agreed to register an account. Online informed consent was obtained from all participants. This study was approved by the Public Health School Medical Center and all experiments were performed in accordance with the approved guidelines and regulations. In IDQC, all subjects were asked to complete an online demographic questionnaire on age, gender, income (three groups: 1 ≤ 2000 yuan per month; 2 = 2000–5000 yuan per month; 3 ≥ 5000 yuan per month.), education level (three groups: 1 = under college, 2 = bachelor, 3 = master or doctor), labor strength (three groups: 1 = light, whose jobs had light physical activity, such as office staff, teachers, shop assistants and so on; 2 = medium, whose jobs had regular physical activity, such as doctors, students and so on; 3 = heavy, whose jobs had heavy physical activity, such as farmers, building workers and so on.), physical exercise (three groups: 1 ≤ 10 h per week; 2 = 10–20 h per week; 3 ≥ 20 h per week.) smoking (three groups, 1 = current smokers and 2 = non-smokers (less than once per month during the past year) 3 = quit smoking (longer than 6 months)) and alcohol use (1 = current drinkers and 2 = non-drinkers (less than once per month during the past year), 3 = quit drinking (longer than 6 months)).

Of all recruited participants, 229 were excluded for incomplete information on the IDQC, extreme daily energy intake (<800 kcal (3349 kJ) or >5000 kcal (20,934 kJ) for males; <600 kcal (2512 kJ) or >4000 kcal (16,747 kJ) for females), diabetes diagnosis or being on a diet in the past 6 months. Finally, 948 participants were included in our study. A sample size of 237 in each group will be sufficient to detect a difference of 1.5 cm in WC between quartiles of dietary BCAA ratio while the standard deviation was 7.8 cm in our overall population, at 84% power and 5% level of significance. This sample size also allows us to detect 1.0 kg/m^2^ difference in BMI, at 99% power and 5% level of significance.

**Figure 1:**
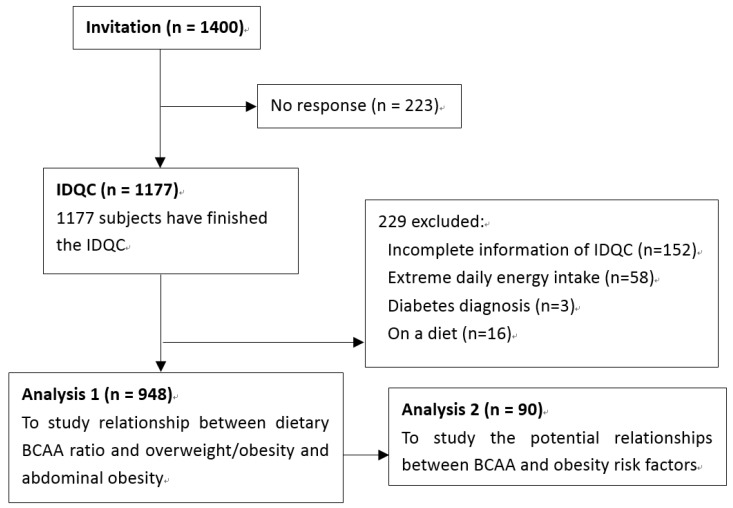
Study design and the flow of participants.

To explore possible mechanisms of the anti-obesity effect of BCAA, 90 subjects were randomly selected to perform a subgroup study. Except for drinking and smoking habits, there is no difference between included participants and excluded participants. Characteristics were presented in Table S3. An oral glucose tolerance test (OGTT) was performed and serum triglycerides (TG), total cholesterol (TC), high density lipoprotein (HDL), low density lipoprotein (LDL), leptin (LEP), adiponectin (ADPN), tumor necrosis factor alpha (TNF-α), interleulin-6 (IL-6) and C-reactive protein (CRP) were also examined. The whole study design is shown in Figure 1.

### 2.4. Dietary Nutrient Intake Calculation

All included participants also completed the IDQC for past 4 months. In the IDQC, commonly eaten foods were divided into 16 categories (including: grains; potatoes; beans and bean products; vegetables; fungus; fruits; seeds and nuts; livestock; poultry; daily; eggs; fish; snacks; sweet foods; condiment and beverage). Each participant had to choose the frequency and amount of each kind of food under each food category. For each kind of food, participants had to choose one of eight options of intake frequency: never (less than 1 time/month), 1–3 times/month, 1 time/week, 2–3 times/week, 4–5 times/week, 1 time/day, 2 times/day, 3 or more than 3 times/day. For most kinds of food, such as apple, carrot, pork, chicken and beef, participants were asked to choose the intake amount of each time from six options: <1 liang (a Chinese traditional food weight unit, 1 liang = 50 g), 2–3 liang, 4–5 liang, 6–7 liang, 8–9 liang, or more than 1 kg. Small-portion food, such as shallot, ginger and garlic, was counted as less than 10 g, 20–30 g, 40–50 g, 60–70 g, 80–90 g, or more than 100 g. Beverages were counted in bottles (550 mL/bottle). Average daily intakes of each kind of food were then calculated. The nutrient content of each food was derived from the China Food Composition Tables [20]. Intake of dietary BCAA and other nutrients were then calculated by IDQC. Ratios of isoleucine, leucine, valine and BCAA were defined as their percentage of total amino acid intake.

### 2.5. Anthropometric Measurements

Height, weight, waist circumference (WC), systolic blood pressure (SBP) and diastolic blood pressure (DBP) of participants were measured by well-trained examiners. Participants were asked to wear light, thin clothing and no shoes. Body weight and height were measured to the nearest 0.1 kg and 0.1 cm. Blood pressure was measured using a standard mercury sphygmomanometer after a 10 min rest in a sitting position. Blood pressure was measured three times and the mean values were calculated. WC was measured midway between the lowest rib and the iliac crest with a flexible anthropometric tape on the horizontal plane with the participant in standing position. WC was measured to the nearest 0.1 cm. WC was measured three times and the mean values were used for subsequent statistical analysis.

### 2.6. Definition of Overweight/Obesity and Abdominal Obesity

BMI was defined as weight in kilograms divided by the square of height in meters (kg/m^2^). The BMI cut-off points in Chinese subjects for overweight (24 ≤ BMI < 28 kg/m^2^) and obesity (BMI ≥ 28 kg/m^2^) were used in our study to define these states [11]. Abdominal obesity was defined when WC ≥ 85 cm in males, and WC ≥ 80 cm in females, according to the 2006 Guidelines on Preservation and Control Overweight and Obesity in Chinese Adults classification [21]. Overweight/obesity plus abdominal obesity was defined as when BMI ≥ 24 kg/m^2^ in both genders, and WC ≥ 85 cm in males, WC ≥ 80 cm in females.

### 2.7. Laboratory Measurements

Ninety participants were instructed to fast for 12 h and fasting blood samples were collected between 8:00 and 9:00 a.m. in the morning. Then, each participant was asked to take 75 g glucose and blood samples were drawn 2 h later. Blood samples were centrifuged at 3000 rpm for 15 min to obtain serum and serum were stored at −80 °C. Fasting blood glucose (FBG), 2 h postprandial glucose (2 h-PG), serum TG, TC, HDL and LDL were determined using a ROCHE Modular P800 Automatic Biochemical Analyzer (Roche Diagnostics, Mannheim, Germany). Serum insulin concentration was measured by ROCHE Elecsys 2010 Chemiluminescence Immune Analyzer (Roche Diagnostics, Mannheim, Germany). Homeostasis model assessment of insulin resistance (HOMA-IR) index was calculated as previously described [22]. Serum LEP, ADPN, TNF-α, IL-6 and CRP concentrations were measured by enzyme linked immunosorbent assay (Elisa Biotech Co., Ltd, Shanghai, China). Coefficient of variations (CVs) of lab measurements were presented in supplemental materials.

### 2.8. Statistical Analysis

Values in the text are means ± standard deviation (SD) unless otherwise indicated. Values for demographic and dietary factors were compared across 4 quartiles of BCAA ratio using one-way analysis of variance (one-way ANOVA), chi-square test or Kruskal-Wallis test. Dietary BCAA ratios of different obesity types were further analyzed by means of analysis of covariance (ANCOVA). Partial correlation analysis was performed to assess the association of BCAA and BMI, WC (adjusting for dietary carbohydrate, fat, protein, cholesterol and fiber intake), FBG, 2 h-PG, TG, TC, HDL, LDL, fasting blood insulin (FBI), HOMA-IR, LEP, ADPN, TNF-α, IL-6 and CRP (adjusting for BMI, dietary carbohydrate, fat, protein, cholesterol and fiber intake). Logistic regression was used to estimate the OR and 95% CI of overweight/obesity and abdominal obesity by quartiles of BCAA intake. Odds ratio (OR) and 95% confidence interval (CI) for overweight status and obesity were calculated in 3 models: model 1, adjusting for age, gender, education, income, labor and exercise status; model 2, additionally adjusting for carbohydrate, fat, protein, cholesterol and fiber intake; and model 3, further adjusting for smoking and drinking habits. All statistical analyses were carried out using SAS software (version 9.1; SAS Institute, Cary, NC, USA). *p* < 0.05 was considered statistically significant.

## 3. Results

### 3.1. Characteristics and Dietary Intakes in the Study Population

The prevalence of overweight/obesity was 12.2% and the prevalence of abdominal obesity was 7.7% among our participants. Characteristics and dietary nutrients intakes of participants are presented by quartile of dietary BCAA ratio in Table 1, respectively. Compared with those in the first quartile of dietary BCAA ratio, participants of the 3rd and 4th quartile were more likely to be male and had lower BMI, WC, and dietary energy percentage of carbohydrates, and had higher dietary energy percentage of protein, fat, dietary cholesterol, fiber and BCAA.

### 3.2. Dietary BCAA Ratios of Overweight/Obesity and Central Obese Participants Were Lower than Healthy Controls

**Figure 2:**
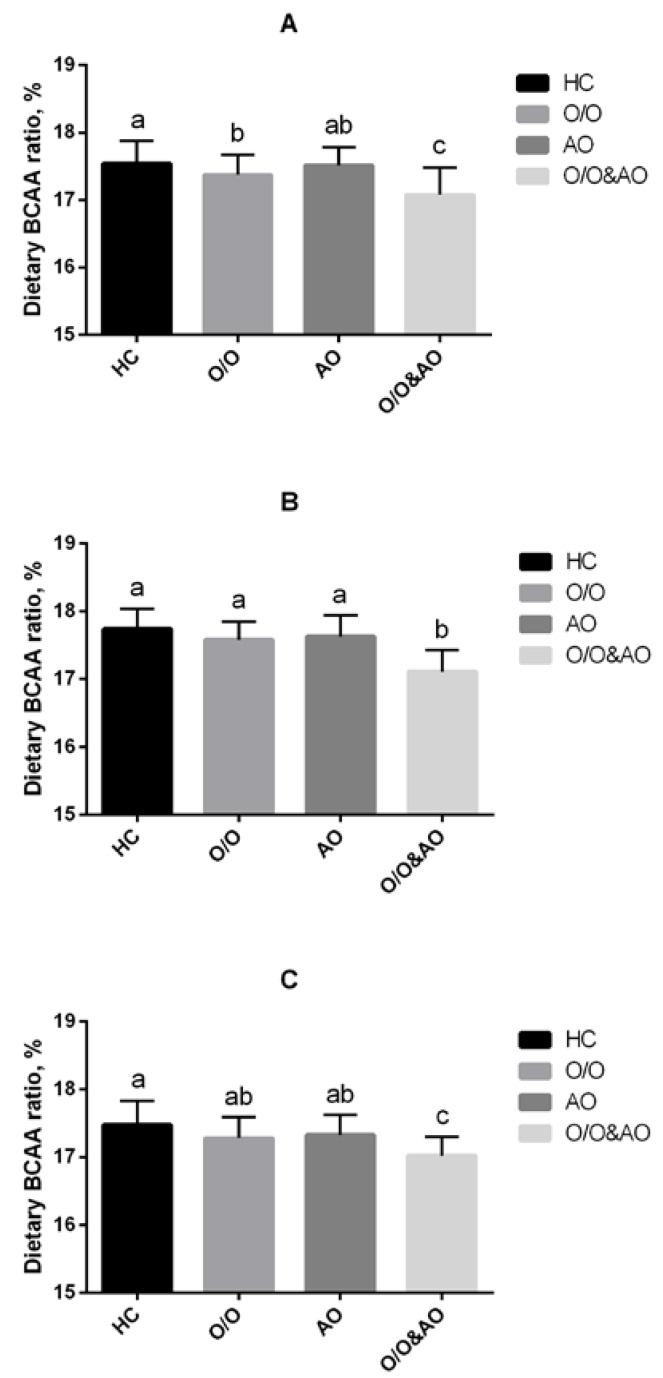
Dietary BCAAs intake of different obese type: HC, healthy control; O/O, overweight/obesity; AO, abdominal obesity; O/O & AO: overweight/obesity & abdominal obesity. (**A**) overall population, *p* < 0.05 between HC and O/O; *p* < 0.05 between O/O and O/O & AO, AO and O/O & AO; *p* < 0.01 between HC & O/O & AO; (**B**) male population, *p* < 0.01 between O/O & AO and other groups; (**C**) female population, *p* < 0.01 between HC and O/O & AO, *p* < 0.05 between O/O and O/O & AO; AO and O/O & AO.

We firstly performed this comparison to find the differences in dietary BCAA ratios among obese subtypes (healthy control (HC); overweight/obesity (O/O); abdominal obesity (AO); overweight/obesity plus abdominal obesity (O/O & AO)). In the whole population, dietary BCAA ratios for O/O, AO, and O/O & AO groups were all significantly lower than that in the HC group (*p* < 0.05). Moreover, the dietary BCAA ratio of the O/O & AO group was even lower than those in O/O and AO groups. After stratifying by gender, similar significant differences still exist in both male and female groups. Confounding factors such as dietary intake of carbohydrate, fat, protein, cholesterol and fiber were controlled in this study (Figure 2).

**Table 1:** Characteristics and dietary intakes of participants by quartile of BCAAs ratio.

Characteristics	Quartiles of BCAAs (% Total Amino Acids Intake)				*p*
	1st (*n* = 237)	2nd (*n* = 237)	3rd (*n* = 237)	4th (*n* = 237)	
	(≤17.07)	(>17.08 and ≤17.54)	(>17.54 and ≤17.94)	(>17.94)	
Age, year	22.5 ± 5.7	23.0 ± 6.3	22.2 ± 5.5	21.4 ± 4.1	<0.01
Gender					<0.001
Male, *n* (%)	43 (18.1)	70 (29.5)	59 (24.9)	87 (36.7)	
Female, *n* (%)	194 (81.9)	167 (70.5)	178 (75.1)	150 (63.3)	
Income per month					NS
<2000 yuan, *n* (%)	10 (4.2)	8 (3.4)	6 (2.5)	6 (2.5)	
2000–5000 yuan, *n* (%)	220 (92.8)	217 (91.6)	221 (93.2)	216 (91.1)	
≥5000 yuan, *n* (%)	7 (3.0)	12 (5.1)	10 (4.2)	15 (6.3)	
Education					<0.05
Under college, *n* (%)	7 (3.0)	6 (2.5)	12 (5.1)	15 (6.3)	
Bachelor, *n* (%)	210 (88.6)	192 (81.0)	199 (84.0)	193 (81.4)	
Master or doctor, *n* (%)	20 (8.4)	39 (16.5)	26 (11.0)	29 (12.2)	
Labor					NS
Light, *n* (%)	23 (9.7)	31 (13.1)	26 (11.0)	14 (5.9)	
Medium, *n* (%)	214 (90.3)	206 (86.9)	211 (89.0)	223 (94.1)	
Exercise					NS
<10 h/week, *n* (%)	103 (43.5)	98 (41.4)	100 (42.2)	96 (40.5)	
10–20 h/week, *n* (%)	107 (45.1)	111 (46.8)	110 (46.4)	103 (43.5)	
≥20 h/week, *n* (%)	27 (11.4)	28 (11.8)	27 (11.4)	38 (16.0)	
Smoking and drinking *					
Current smoker, *n* (%)	1 (0.4)	11 (4.6)	6 (2.5)	7 (3.0)	<0.001
Current drinker, *n* (%)	15 (6.3)	35 (14.8)	31 (13.1)	26 (11.0)	<0.05
Weight, kg	57.8 ± 10.2	59.2 ± 10.8	57.5 ± 9.3	57.5 ± 9.3	NS
BMI, kg/m^2^	21.5 ± 2.7	21.5 ± 2.9	20.7 ± 2.4	20.5 ± 2.2	<0.001
<24 (normal), *n* (%)	199 (84.0)	199 (84.0)	215 (90.7)	219 (92.4)	<0.05
Male, *n* (%)	26 (11.0)	55 (23.2)	51 (21.5)	80 (33.8)	<0.01
Female, *n* (%)	173 (73.0)	144 (60.8)	164 (69.2)	139 (58.6)	<0.05
≥24 (overweight/obesity), *n* (%)	38 (16.0)	38 (16.0)	22 (9.3)	18 (7.6)	<0.05
Male, *n* (%)	17 (7.2)	15 (6.3)	8 (3.4)	7 (3.0)	<0.05
Female, *n* (%)	21 (8.9)	23 (9.7)	14 (5.9)	11 (4.6)	<0.05
WC, cm	73.6 ± 8.2	72.8 ± 8.4	72.1 ± 7.0	71.2 ± 7.3	<0.01
Abdominal obesity, *n* (%)	25 (10.5)	22 (9.3)	13 (5.5)	13 (5.5)	<0.05
Male, *n* (%)	14 (5.9)	10 (4.2)	9 (3.8)	7 (3.0)	<0.05
Female, *n* (%)	11 (4.6)	12 (5.1)	4 (1.7)	6 (2.5)	<0.05
Overweight/obesity & abdominal obesity, *n* (%)	18 (7.6)	17 (7.2)	7 (3.0)	6 (2.5)	<0.05
Male, *n* (%)	11 (4.6)	11 (4.6)	5 (2.1)	3 (1.3)	<0.05
Female, *n* (%)	7 (3.0)	6 (2.5)	2 (0.9)	3 (1.3)	<0.05
SBP, mmHg	112.2 ± 12.5	114.2 ± 10.0	111.8 ± 12.0	113.6 ± 11.4	NS
DBP, mmHg	77.1 ± 10.3	78.4 ± 8.9	76.3 ± 10.3	77.4 ± 8.8	NS
Dietary intakes ^#^					
Energy, kcal/day	2250.3 ± 54.8	2460.5 ± 52.5	2442.6 ± 57.2	2392.9 ± 61.1	<0.01
Total carbohydrate, g/day	373.9 ± 9.5	376.1 ± 8.5	368.0 ± 9.3	353.6 ± 10.0	NS
% energy	64.1 ± 0.7	58.8 ± 0.6	57.7 ± 0.6	57.4 ± 0.7	<0.001
Total fat, g/day	58.9 ± 2.4	75.3 ± 2.5	75.9 ± 2.3	76.0 ± 2.6	<0.001
% energy	22.7 ± 0.6	26.9 ± 0.6	27.7 ± 0.5	28.3 ± 0.6	<0.001
Cholesterol, mg/day	257.5 ± 15.2	405.3 ± 14.9	421.1 ± 15.5	433.5 ± 17.2	<0.001
Fiber, g/day	18.5 ± 0.8	19.6 ± 0.6	17.5 ± 0.6	14.1 ± 0.6	<0.001
Total protein, g/day	75.0 ± 2.1	88.0 ± 2.1	88.9 ± 2.4	85.3 ± 2.5	<0.001
% energy	13.2 ± 0.1	14.3 ± 0.1	14.6 ± 0.1	14.3 ± 0.2	<0.001
Total amino acids, g/day	48.6 ± 1.6	53.6 ± 1.5	52.8 ± 1.8	48.8 ± 1.9	NS
BCAAs	8.1 ± 0.3	9.3 ± 0.3	9.4 ± 0.3	8.9 ± 0.3	<0.01
Isoleucine	2.1 ± 0.1	2.4 ± 0.1	2.4 ± 0.1	2.3 ± 0.1	<0.05
Leucine	3.7 ± 0.1	4.2 ± 0.1	4.3 ± 0.1	4.1 ± 0.2	<0.05
Valine	2.4 ± 0.1	2.7 ± 0.1	2.7 ± 0.1	2.5 ± 0.1	<0.05

BCAAs: branched-chain amino acids; BMI: body mass index; WC: waist circumference; SBP: systolic blood pressure; DBP: diastolic blood pressure. * There is no report of quitting smoking or quitting drinking in our population. *p* Values are for differences across quartiles of BCAA intake; NS: no significant difference across quartiles. ^#^ Data for dietary intakes are mean ± SEM.

### 3.3. Dietary BCAA Ratio Was Inversely Associated with BMI and Waist Circumference in the Study Population

Partial correlation analysis was used (controlled for dietary intake in carbohydrate, fat, protein, cholesterol and fiber). We found negative correlations between dietary isoleucine, leucine, valine, BCAA ratios and BMI (*r* = −0.160, −0.172, −0.109 and −0.197 respectively, all *p* < 0.001) and WC (*r* = −0.113, −0.177, −0.133 and −0.187 respectively, all *p* < 0.001). Because of higher BCAA intake in males (male: 10.5 ± 5.4 g *vs.* female: 8.3 ± 4.1 g, *p* < 0.05), we examined the associations between dietary BCAA ratio and BMI and WC in males and females separately. Significance still exists in both male and female subgroups (Table 2).

**Table 2:** Partial correlation analysis of ratio of BCAAs, isoleucine, leucine, and valine *versus* BMI and waist.

Parameters	BMI		Waist	
	*r*	*p*	*r*	*p*
Overall				
Isoleucine	−0.160	<0.001	−0.113	<0.001
Leucine	−0.172	<0.001	−0.177	<0.001
Valine	−0.109	<0.01	−0.133	<0.01
BCAAs	−0.197	<0.001	−0.187	<0.001
Male				
Isoleucine	−0.279	<0.001	−0.233	<0.001
Leucine	−0.224	<0.001	−0.209	<0.001
Valine	−0.187	<0.05	−0.115	<0.05
BCAAs	−0.278	<0.001	−0.256	<0.001
Female				
Isoleucine	−0.124	<0.001	−0.113	<0.001
Leucine	−0.182	<0.001	−0.257	<0.001
Valine	−0.110	<0.05	−0.102	<0.05
BCAAs	−0.196	<0.001	−0.254	<0.001

BCAAs, branched-chain amino acids; BMI, body mass index; WC, waist circumference. Adjusting for dietary carbohydrate, fat, protein, cholesterol and fiber intake.

### 3.4. Associations between BCAA Ratio and Prevalence of Overweight/Obesity, and Abdominal Obesity

With adjustment for potential dietary and non-dietary cofounders (age, gender, income, education, labor, physical exercise, dietary carbohydrate, fat, protein, cholesterol and fiber intake and smoking or drinking status), BCAA intake was inversely related to overweight/obesity and abdominal obesity. Compared with the 1st quartile, the multivariable-adjusted ORs for the 3rd and 4th quartile of dietary BCAA ratio were 0.508 (95% CI = 0.265–0.972) and 0.389 (95% CI = 0.193–0.783) for overweight/obesity and 0.351 (95% CI = 0.145–0.845) and 0.376 (95% CI = 0.161–0.876) for abdominal obesity, respectively (all *p* < 0.05). Because of the higher intake of BCAA in males, we examined the relationship between dietary BCAA ratio and overweight/obesity or abdominal obesity in males and females separately. As a result, the relationship is stronger in males than females (Table 3).

**Table 3:** Multivariable-adjusted OR and 95% CI of overweight/obesity and abdominal obesity by quartile of BCAAs ratio.

BCAA Quartiles	Quartile 1	Quartile 2	Quartile 3	Quartile 4
**Overweight/obesity**				
All				
Crude	1	1.000 (0.612, 1.633)	0.536 (0.306, 0.938) *	0.430 (0.238, 0.779) **
Model 1	1	0.978 (0.564, 1.697)	0.501 (0.268, 0.937) *	0.403 (0.208, 0.780) **
Model 2	1	0.958 (0.543, 1.688)	0.509 (0.265, 0.976) *	0.392 (0.195, 0.789) **
Model 3	1	0.963 (0.546, 1.700)	0.508 (0.265, 0.972) *	0.389 (0.193, 0.783) **
Male				
Crude	1	0.468 (0.218, 1.006)	0.185 (0.073, 0.469) **	0.161 (0.061, 0.430) **
Model 1	1	0.542 (0.229, 1.280)	0.255 (0.096, 0.678) **	0.186 (0.063, 0.551) **
Model 2	1	0.594 (0.242, 1.454)	0.303 (0.107, 0.853) *	0.206 (0.064, 0.659) **
Model 3	1	0.525 (0.210, 1.312)	0.264 (0.093, 0.752) *	0.170 (0.053, 0.550) **
Female				
Crude	1	0.883 (0.449, 1.733)	0.726 (0.359, 1.470)	0.469 (0.213, 1.034)
Model 1	1	0.696 (0.344, 1.453)	0.635 (0.298, 1.356)	0.404 (0.174, 0.941) *
Model 2	1	0.644 (0.306, 1.354)	0.529 (0.242, 1.157)	0.310 (0.125, 0.733) *
Model 3	1	0.628 (0.298, 1.325)	0.533 (0.243, 1.165)	0.316 (0.127, 0.789) *
**Abdominal obesity**				
All				
Crude	1	0.872 (0.477, 1.594)	0.496 (0.247, 0.996) *	0.494 (0.246, 0.991) *
Model 1	1	0.704 (0.350, 1.414)	0.343 (0.147, 0.801) *	0.368 (0.166, 0.818) *
Model 2	1	0.710 (0.348, 1.450)	0.349 (0.147, 0.832) *	0.379 (0.165, 0.868) *
Model 3	1	0.671 (0.325,1.385)	0.351 (0.145, 0.845) *	0.376 (0.161, 0.876) *
Male				
Crude	1	0.464 (0.205, 1.048)	0.417 (0.181, 0.959) *	0.101 (0.028, 0.359) **
Model 1	1	0.441 (0.169, 1.150)	0.426 (0.164, 1.106)	0.084 (0.018, 0.397) **
Model 2	1	0.433 (0.164, 1.144)	0.417 (0.153, 1.136)	0.079 (0.016, 0.380) **
Model 3	1	0.351 (0.127, 0.969) *	0.383 (0.138, 1.067)	0.066 (0.013, 0.333) **
Female				
Crude	1	1.455 (0.541, 3.916)	0.565 (0.162, 1.965)	0.710 (0.221, 2.282)
Model 1	1	1.256 (0.443, 3.556)	0.538 (0.150, 1.930)	0.685 (0.204, 2.302)
Model 2	1	1.331 (0.456, 3.883)	0.582 (0.155, 2.182)	0.730 (0.202, 2.643)
Model 3	1	1.299 (0.445, 3.797)	0.578 (0.154, 2.171)	0.748 (0.206, 2.713)

BCAAs, branched-chain amino acids. Model 1: adjusting for age, gender, education, income, labor, exercise. Model 2: model 1 + carbohydrate, fat, protein, cholesterol and fiber intake were adjusted. Model 3: model 2 + smoking, drinking were adjusted. * *p* < 0.05; ** *p* < 0.01 compared with the 1st quartile.

### 3.5. Dietary BCAA Ratio was Negatively Associated with 2 h-PG in Overall and Male Young Adults

Partial correlation analysis was used after BMI, dietary intake of carbohydrate, fat, protein, cholesterol and fiber were controlled. We found negative correlations between BCAA ratios and 2 h-PG in overall, male and female populations (*r* = −0.316, −0.373, −0.211, all *p* < 0.05). In the male population, ratio of dietary leucine was negatively associated with FBG (*r*= −0.375, *p* < 0.05). However, there is no significant correlation between dietary BCAA ratio and FBG, FBI and HOMA-IR (Table 4).

**Table 4:** Partial correlation analysis of ratio of BCAAs, isoleucine, leucine, and valine and metabolic parameters.

Parameters	FBG		2 h-PG		FBI		HOMA-IR		TG		TC		HDL		LDL	
	*r*	*p*	*r*	*p*	*r*	*p*	*r*	*p*	*r*	*p*	*r*	*p*	*r*	*p*	*r*	*p*
Overall																
Isoleucine	−0.089	0.216	−0.102	0.109	−0.096	0.671	−0.131	0.213	−0.082	0.356	−0.136	0.712	0.146	0.181	−0.079	0.471
Leucine	−0.128	0.125	−0.157	0.129	−0.135	0.289	−0.194	0.289	−0.121	0.331	−0.077	0.893	0.067	0.540	−0.193	0.077
Valine	−0.119	0.365	−0.107	0.168	−0.097	0.531	−0.102	0.547	−0.078	0.677	−0.010	0.972	0.040	0.715	0.010	0.926
BCAA	−0.205	0.066	−0.316	0.037	−0.182	0.139	−0.221	0.095	−0.098	0.543	0.061	0.698	0.026	0.810	0.031	0.781
Male																
Isoleucine	−0.017	0.618	−0.132	0.124	−0.101	0.457	−0.221	0.210	−0.079	0.652	−0.128	0.589	0.279	0.081	0.100	0.539
Leucine	−0.375	0.017	−0.202	0.055	−0.199	0.382	−0.139	0.472	−0.211	0.219	−0.022	0.940	−0.099	0.544	−0.122	0.400
Valine	−0.132	0.203	−0.112	0.106	−0.163	0.291	−0.178	0.313	−0.035	0.864	−0.058	0.921	0.243	0.130	−0.012	0.944
BCAA	−0.252	0.051	−0.373	0.001	−0.178	0.117	−0.213	0.117	−0.119	0.421	−0.079	0.689	0.161	0.322	−0.067	0.682
Female																
Isoleucine	−0.277	0.083	−0.068	0.279	−0.033	0.981	−0.067	0.668	−0.098	0.443	−0.071	0.783	0.101	0.535	−0.150	0.357
Leucine	−0.118	0.468	−0.129	0.158	−0.181	0.421	−0.126	0.479	−0.112	0.312	−0.099	0.769	−0.002	0.990	0.158	0.332
Valine	−0.258	0.108	−0.107	0.235	−0.102	0.765	−0.132	0.348	−0.084	0.547	−0.035	0.891	−0.072	0.660	0.197	0.222
BCAA	−0.246	0.073	−0.211	0.045	−0.168	0.235	−0.186	0.138	−0.093	0.455	−0.067	0.621	−0.046	0.780	−0.061	0.707

FBG, fasting blood glucose; 2 h-PG, 2 h postprandial glucose; FBI, fasting blood insulin; HOMA-IR, homeostasis model assessment of insulin resistance; TG, triglycerides; TC, total cholesterol; HDL, high density lipoprotein; LDL, low density lipoprotein. Adjusting for BMI, dietary carbohydrate, fat, protein, cholesterol and fiber intake.

### 3.6. Dietary BCAA Ratio Was Positively Associated with Serum LEP and ADPN in Overall and Male Adults

Partial correlation analysis was used after BMI, dietary intake of carbohydrate, fat, protein, cholesterol and fiber were controlled. We found positive correlations between BCAA ratios and serum LEP (*r* = 0.211 and 0.235, for overall and males, all *p* < 0.05) and ADPN (*r* = 0.231, 0.236 and 0.218 for overall, males and females, respectively, all *p* < 0.05) and negative correlations between BCAA ratios and serum CRP (*r* = −0.229, −0.275 and −0.219 for overall, males and females, all *p* < 0.05). However, there is no significant correlation between dietary BCAA ratio and serum TNF-α and IL-6 (Table 5).

**Table 5:** Partial correlation analysis of ratio of BCAAs, isoleucine, leucine, and valine and level of inflammation.

Parameters	LEP		ADPN		TNF-α		IL-6		CRP	
	*r*	*p*	*r*	*p*	*r*	*p*	*r*	*p*	*r*	*p*
Overall										
Isoleucine	0.113	0.331	0.108	0.365	−0.123	0.222	−0.117	0.421	−0.137	0.192
Leucine	0.192	0.109	0.212	0.099	−0.162	0.169	−0.124	0.265	−0.256	0.043
Valine	0.098	0.421	0.088	0.643	−0.102	0.289	−0.101	0.348	−0.127	0.231
BCAA	0.211	0.045	0.231	0.042	−0.191	0.055	−0.115	0.281	−0.229	0.035
Male										
Isoleucine	0.147	0.264	0.131	0.273	−0.131	0.231	−0.122	0.487	−0.161	0.181
Leucine	0.201	0.052	0.233	0.047	−0.139	0.372	−0.098	0.544	−0.281	0.023
Valine	0.111	0.389	0.079	0.586	−0.104	0.313	−0.137	0.286	−0.116	0.312
BCAA	0.235	0.03	0.236	0.021	−0.193	0.107	−0.117	0.331	−0.275	0.031
Female										
Isoleucine	0.102	0.412	0.117	0.452	−0.087	0.628	−0.111	0.367	−0.145	0.211
Leucine	0.189	0.131	0.178	0.142	−0.116	0.437	−0.145	0.287	−0.236	0.030
Valine	0.102	0.423	0.099	0.398	−0.141	0.333	−0.094	0.621	−0.133	0.233
BCAA	0.198	0.071	0.218	0.044	−0.171	0.102	−0.136	0.221	−0.219	0.040

LEP, leptin; ADPN, adiponectin; TNF-α, tumor necrosis factor alpha; IL-6, interleulin-6; CRP, C-reactive protein. Adjusting for BMI, dietary carbohydrate, fat, protein, cholesterol and fiber intake.

### 3.7. Dietary BCAA Ratio Was Positively Associated with Serum LEP and ADPN in Overall and Male Adults

Partial correlation analysis was used after BMI and dietary intake of carbohydrates, fat, protein, cholesterol and fiber were controlled. We found positive correlations between BCAA ratios and serum LEP (*r* = 0.211 and 0.235, for overall and males, all *p* < 0.05) and ADPN (*r* = 0.231, 0.236 and 0.218 for overall, males and females respectively, all *p* < 0.05) and negative correlations between BCAA ratios and serum CRP (*r* = −0.229, −0.275 and −0.219 for overall, males and females, all *p* < 0.05). However, no significant correlations between dietary BCAA ratio and serum TNF-α and IL-6 were observed (Table 5).

## 4. Discussion

In this internet-based cross-sectional study, the ratio of dietary BCAA intake was inversely associated with prevalence of obesity, postprandial glucose and inflammation in young northern Chinese population, which was firstly found using a FFQ study.

Several previous animal studies may indirectly support this finding. Belobrajdic *et al.* reported that higher dietary protein could reduce the energy intake and body fat of male Wistar rats, and increasing the dietary density of whey protein concentrate is more effective than red meat in reducing bodyweight gain [23]. As we know, whey protein contains a greater proportion of BCAA than meat. It is also reported that an increase in dietary leucine intake could produce some health benefits, such as restriction of diet-induced weight gain, hyperglycemia and hypercholesterolemia in high-fat diet mice [24]. Thus, BCAA or leucine could be suggested as potential candidates for maintenance of body weight or improvement of the metabolic profile.

In some population studies, randomized controlled trials reported that diets high in protein (rich of BCAA) may lower body weight in obese individuals [25,26,27]. One relevant study of 25 wrestlers has reported that the combination of moderate energy restriction and BCAA supplementation was found to reduce body weight and visceral adipose tissue in a short-term intervention. Those consuming the high-BCAA diet had the greatest reduction of body weight (−4.0 kg; *p* < 0.05) and body fat percentage (−17.3%; *p* < 0.05) [3]. To our knowledge, only one study reported that higher dietary BCAA intake was associated with lower prevalence of overweight/obesity in the general population in Eastern Asian and Western countries [10]. However, this study, based on 24-h recall dietary survey, cannot represent long-term habitual food intake. We still do not know the exact effects of long-term habitual BCAA intake on the body weight and metabolic profile of healthy subjects.

In this study, long-term habitual BCAA intake was inversely associated with BMI and WC in the general population, which might be explained by several potential mechanisms as follows. Leucine, the main component of BCAA (half of dietary BCAA), serves not only as a component for protein synthesis but is also a potent activator of the mammalian target of rapamycin (mTOR), a serine/threonine kinase involved in many cellular processes, including protein synthesis, cell growth and metabolism [28,29]. Central administration of leucine could increase hypothalamic mTOR signaling and decrease food intake and body weight [30]. Moreover, leptin, an adipose-derived hormone regulating energy intake and expenditure [31], could also be stimulated by leucine supplementation, and leptin levels were reduced by 40% in rats provided a leucine-deficient meal [32]. In our study, we observed a positive association between the ratio of dietary BCAA and serum leptin. Leucine supplementation also could decreased diet-induced obesity by increasing resting energy expenditure associated with increased uncoupling protein 3 (UCP3) expression in thermo-genic tissues in mice [24]. Our finding may be partially explained by these mechanisms.

Overweight and obesity are always accompanied by abnormal postprandial metabolism. Therefore, maintaining postprandial metabolism is of significant importance for weight control. It has been reported that BCAA could improve glucose tolerance. In animal studies, increased leucine intake could improve glucose metabolism, reduce diet-induced insulin resistance and lower plasma glucagon levels and hepatic glucose-6-phosphatase expression [24]. Moreover, leucine may also affect glucose metabolism by stimulating insulin release from the pancreas [33,34,35]. Isoleucine administration in rats could stimulate both glucose uptake in the muscle and whole body glucose oxidation, depressing glucose genesis in the liver and thereby leading to a hypoglycemic effect [36,37]. One population-based study suggested that higher intake of BCAA might be associated with a lower risk for diabetes. In female, valine intake was significantly marginally associated with the risk of diabetes; the hazard ratio for the highest tertile *vs.* the lowest was 0.61 (0.39–0.94; *p* = 0.03) [38]. In our study, we found a higher dietary BCAA ratio was associated with lower 2 h-PG in overall, male and female groups. These results indicated that dietary BCAA might induce a higher level of glucose oxidation in the general population, which was consistent with previous animal studies [24]. However, a single component of BCAA did not show significant effect on postprandial blood glucose, and this indicated a synergistic effect from these amino acids. However, no significant associations between dietary BCAA ratio and FBG, FBI, HOMA-IR, TG, TC, HDL and LDL were observed in the non-obese population. Limited sample size may influence this effect and further studies are needed to confirm this association.

Obesity is considered to be accompanied by a chronic low-grade inflammatory state that contributes to the occurrence of many chronic diseases. Therefore, the suppression of low-grade chronic pro-inflammation is an effective strategy for reducing the risk of obesity-related diseases. BCAA also plays a vital role in the progress of anti-inflammation. Oral administration of BCAA granules decreased highly sensitive CRP in HCV-positive patients with liver cirrhosis [39]. Leucine supplementation also improved adiponectin in a high-fat diet rat model [40]. Zemel *et al.* also reported that the anti-inflammatory biomarker adiponectin was also increased by supplementation of leucine and pyridoxine in obese individuals [41]. These studies also support our results. However, no significant associations were observed between dietary BCAA and TNF-α or IL-6; a limited dose from daily meals may explain this insignificance.

For the gender difference in BCAA in our study, we found that the association between the ratio of dietary BCAA and abdominal obesity is much stronger in males. Several potential mechanisms might account for this finding. Firstly, higher BCAA intake was observed in male participants than female participants in our population (10.5 g in male and 8.3 g in female), and more BCAA may exert stronger effects. Secondly, testosterone secretion can be stimulated by BCAA supplementation [42], and then increases lean body mass and decreases fat mass of adults [43]. Differences of serum testosterone levels between males and females may also favor the potentially beneficial effects of dietary BCAA.

The cross-sectional design is a limitation of this study. It cannot confirm the causal relationship between BCAA ratio and development of overweight/obesity or abdominal obesity. Therefore, cohort studies for verification of this issue are needed. Moreover, as the current study was only performed in a northern Chinese population, further studies are needed to verify this association in the southern Chinese population. In terms of the strengths of the study, this is the first web-based FFQ study that has investigated this issue. Compared with traditional FFQs, the reference images of different weights of each food in our IDQC contribute to a more precise estimation of food intake. The internet and mobile devices make our IDQC a convenient tool for the dietary survey. Our study adds evidence supporting the inverse associations between dietary BCAA ratios and prevalence of overweight/obesity and abdominal obesity, possibly through the improvements of metabolic profile and inflammation by BCAA. Further studies are needed to verify the causal association and explore the potential mechanism.

## 5. Conclusions

In conclusion, we found higher ratio of dietary BCAA intake is inversely associated with prevalence of overweight/obesity, abdominal obesity, postprandial glucose tolerance and status of inflammation in young northern Chinese adults. However, long-term cohort studies are needed to verify if BCAA could prevent the incidence of overweight/obesity and abdominal obesity.

## Supplementary Materials

The following are available online at http://www.mdpi.com/2072-6643/7/11/5486/s1, Table S1: STROBE checklist of our study (1), Table S2: STROBE checklist of our study (2), Table S3: STROBE checklist of our study (3).
