# Supplementary material for: The Ratio of Dietary Branched-Chain Amino Acids is Associated with a Lower Prevalence of Obesity in Young Northern Chinese Adults: An Internet-Based Cross-Sectional Study
Source: Nutrients. 2015 Nov 18;7(11):9573–89. doi: 10.3390/nu7115486 (PMC4663614; doi:10.3390/nu7115486)
Supplement: Supplementary file 1 [file nutrients-07-05486-s001.docx]

Supplementary Materials: The Ratio of Dietary Branched-Chain Amino Acids is Associated with a Lower Prevalence of Obesity in Young Northern Chinese Adults: an Internet-Based Cross-Sectional Study

Yan-Chuan Li ^1^, Ying Li ^1^, Li-Yan Liu ^1^, Yang Chen ^1^, Tian-Qi Zi ^1^, Shan-Shan Du ^1^, Yong-Shuai Jiang ^2^, Ren-Nan Feng ^1,^* and Chang-Hao Sun ^1,^*

STROBE Statement—Checklist of items that should be included in reports of ***cross-sectional studies*.**

**Table S1.** STROBE checklist of our study (1).

| **Item** | Item No. | Recommendation | Self-Checking Result |
| --- | --- | --- | --- |
| **Title and abstract** | 1 | (*a*) Indicate the study’s design with a commonly used term in the title or the abstract | Yes, study design were indicated with commonly used term in the title and abstract. |
|  |  | (*b*) Provide in the abstract an informative and balanced summary of what was done and what was found | Yes, these details were provided in the abstract. |
| Introduction | | |  |
| Background/rationale | 2 | Explain the scientific background and rationale for the investigation being reported | Yes, the scientific background and rationale of the investigation were explained in introduction part. |
| Objectives | 3 | State specific objectives, including any prespecified hypotheses | Yes, objectives of this study and prespecified hypotheses were stated in introduction part. |
| Methods | | |  |
| Study design | 4 | Present key elements of study design early in the paper | Yes, key elements of study design were presented in methods section of the manuscript. |
| Setting | 5 | Describe the setting, locations, and relevant dates, including periods of recruitment, exposure, follow-up, and data collection | Yes, the setting, locations, and relevant dates were described in methods section of the manuscript. |
| Participants | 6 | (*a*) Give the eligibility criteria, and the sources and methods of selection of participants | Yes, criteria, sources and methods of selection of participants were given in methods part of manuscript. |
| Variables | 7 | Clearly define all outcomes, exposures, predictors, potential confounders, and effect modifiers. Give diagnostic criteria, if applicable | Yes, all outcomes, exposures and potential confounders were clearly defined, please check methods part of manuscript. |
| Data sources/measurement | 8 * | For each variable of interest, give sources of data and details of methods of assessment (measurement). Describe comparability of assessment methods if there is more than one group | Yes, sources of data and details of assessment methods were given for interested variables. Assessment methods were consistent among all participants. |
| Bias | 9 | Describe any efforts to address potential sources of bias | Yes, we addressed potential bias and described our efforts. |
| Study size | 10 | Explain how the study size was arrived at | Yes, we explained how the study size was arrived. |
| Quantitative variables | 11 | Explain how quantitative variables were handled in the analyses. If applicable, describe which groupings were chosen and why | Yes, we explained how quantitative variables were handled in the analyses. |

* Give information separately for exposed and unexposed groups.

**Table S2.** STROBE checklist of our study (2).

| **Item** | **Item No.** | **Recommendation** | **Self-Checking Result** |
| --- | --- | --- | --- |
| **Methods** | | |  |
| Statistical methods | 12 | (*a*) Describe all statistical methods, including those used to control for confounding | Yes, all statistical methods were described, including adjustment of potential confounders. |
|  |  | (*b*) Describe any methods used to examine subgroups  and interactions | Yes, we described methods to examine subgroups. |
|  |  | (*c*) Explain how missing data were addressed | Yes, please check manuscript page 4 line 106. |
|  |  | (*d*) If applicable, describe analytical methods taking account of sampling strategy | Yes, we described sampling strategy in the manuscript. |
|  |  | (*e*) Describe any sensitivity analyses | Not applicable in our study. |
| Results | | |  |
| Participants | 13 * | (*a*) Report numbers of individuals at each stage of study—e.g., numbers potentially eligible, examined for eligibility, confirmed eligible, included in the study, completing follow-up, and analysed | Yes, numbers of individuals at each stage of study were reported in flow diagram of the manuscript (Figure 1). |
|  |  | (*b*) Give reasons for non-participation at each stage | Yes, details were shown in Figure 1. |
|  |  | (*c*) Consider use of a flow diagram | Yes, flow diagram was used in our manuscript. |
| Descriptive data | 14 * | (*a*) Give characteristics of study participants  (e.g., demographic, clinical, social) and information  on exposures and potential confounders | Yes, characteristics of study participants were presented in Table 1. |
|  |  | (*b*) Indicate number of participants with missing data for each variable of interest | No missing data for interested variables. |
| Outcome data | 15 * | Report numbers of outcome events or summary measures | Yes, I confirm that these details were presented in Table 1. |

* Give information separately for exposed and unexposed groups.

**Table S3.** STROBE checklist of our study (3).

| **Item** | **Item No.** | **Recommendation** | **Self-Checking Result** |
| --- | --- | --- | --- |
| **Results** | | |  |
| Main results | 16 | (*a*) Give unadjusted estimates and, if applicable, confounder-adjusted estimates and their precision (e.g., 95% confidence interval). Make clear which confounders were adjusted for and why they were included | Yes, details were presented in Table 3. |
|  |  | (*b*) Report category boundaries when continuous variables were categorized | Yes, details were presented in Table 1. |
|  |  | (*c*) If relevant, consider translating estimates of relative risk into absolute risk for a meaningful time period | Not applicable |
| Other analyses | 17 | Report other analyses done—e.g., analyses of subgroups and interactions, and sensitivity analyses | Not applicable |
| Discussion | | |  |
| Key results | 18 | Summarise key results with reference to study objectives | Yes, key results with reference to study objectives were summarised in the first paragraph of discussion part of the manuscript. |
| Limitations | 19 | Discuss limitations of the study, taking into account sources of potential bias or imprecision. Discuss both direction and magnitude of any potential bias | Yes, limitations of the study were discussed in discussion part of the manuscript. |
| Interpretation | 20 | Give a cautious overall interpretation of results considering objectives, limitations, multiplicity of analyses, results from similar studies, and other relevant evidence | Yes, a cautious overall interpretation of results considering objectives, limitations, multiplicity of analyses, results from similar studies, and other relevant evidence were given in the manuscript. |
| Generalisability | 21 | Discuss the generalisability (external validity) of the study results | Yes, the validity of the study was discussed. |
| Other information | | |  |
| Funding | 22 | Give the source of funding and the role of the funders for the present study and, if applicable, for the original study on which the present article is based | Yes, source of funding were provided at the end of the manuscript. |

* Give information separately for exposed and unexposed groups; Note: An Explanation and Elaboration article discusses each checklist item and gives methodological background and published examples of transparent reporting. The STROBE checklist is best used in conjunction with this article (freely available on the Web sites of PLoS Medicine at http://www.plosmedicine.org/, Annals of Internal Medicine at http://www.annals.org/, and Epidemiology at http://www.epidem.com/). Information on the STROBE Initiative is available at www.strobe-statement.org.
